# Huachansu triggers mitochondrial apoptosis and ER stress to inhibit hepatocellular carcinoma progression

**DOI:** 10.1007/s44446-026-00089-9

**Published:** 2026-07-13

**Authors:** Ximeng Li, Qiuying Yan, Qibiao Wu, Dan Dong, Runjing Zhang, Qinghai Meng, Changliang Xu, Yueyang Lai, Jiani Tan, Chengtao Yu, Liu Li, Weixing Shen, Qianjun Chen, Haibo Cheng, Dongdong Sun

**Affiliations:** 1 The Second Clinical College of Guangzhou University of Chinese Medicine, Guangzhou, China; 2 China Resources Sanjiu Medical & Pharmaceutical Co., Ltd., Shenzhen, China; 3 Jiangsu Collaborative Innovation Center of Traditional Chinese Medicine in Prevention and Treatment of Tumor, Nanjing University of Chinese Medicine, Nanjing, China; 4 Macau University of Science and Technology, Avenida da Universidade, Macau, China; 5 The First Clinical Medical College, Nanjing University of Chinese Medicine, Nanjing, China; 6 State Key Laboratory of Traditional Chinese Medicine Syndrome, The Second Affiliated Hospital of Guangzhou University of Chinese Medicine, Guangzhou, Guangdong China; 7 Chinese Medicine Guangdong Laboratory, Hengqin, Guangdong China; 8 Guangdong Provincial Hospital of Chinese Medicine, Guangzhou, China

**Keywords:** Hepatocellular carcinoma, Huachansu, Mitochondrial damage, Endoplasmic reticulum stress, Apoptosis

## Abstract

Huachansu, derived from the dried skin glands of the Chinese toad (*Bufo bufo gargarizans*), has been used in traditional Chinese medicine for its anti-tumor properties, particularly in hepatocellular carcinoma (HCC). However, its molecular mechanisms remain unclear. This study investigates Huachansu’s therapeutic potential by focusing on mitochondrial apoptosis and endoplasmic reticulum (ER) stress pathways. *In vitro* studies on HepG2 cells revealed that Huachansu (48–96 mg/mL for 24 h) disrupted mitochondrial function by elevating reactive oxygen species (ROS) generation (~ 83% and ~ 208% increase at 48 and 96 mg/mL, respectively) and reducing membrane potential. Molecular analyses showed upregulated pro-apoptotic Bax and ER stress effector CHOP alongside downregulated anti-apoptotic Bcl-2 and caspase family proteins, indicating PERK-ATF4 pathway activation. In vivo investigations using orthotopic and xenograft HCC mouse models demonstrated significant tumor volume (~ 25% at 2 g/kg and ~ 39% at 4 g/kg) and weight reduction with Huachansu treatment (2–4 g/kg/day, oral gavage for 18–21 days). Hepatic function improvements were evidenced by decreased serum ALT, AST, and LDH levels alongside increased superoxide dismutase (SOD) activity. Histopathological analyses confirmed reduced tumor progression and organ toxicity, while inflammatory cytokine profiling revealed diminished pro-inflammatory mediators. Mechanistically, Huachansu activated ER stress markers (PERK, ATF4) in tumor tissues without inducing systemic toxicity. These findings demonstrate that Huachansu exerts potent anticancer effects in HCC through concurrent induction of mitochondrial damage and ER stress activation. This study provides a mechanistic basis for Huachansu’s traditional use and highlights its potential as a promising therapeutic agent for HCC treatment.

**Graphical Abstract:**

**Figure:**
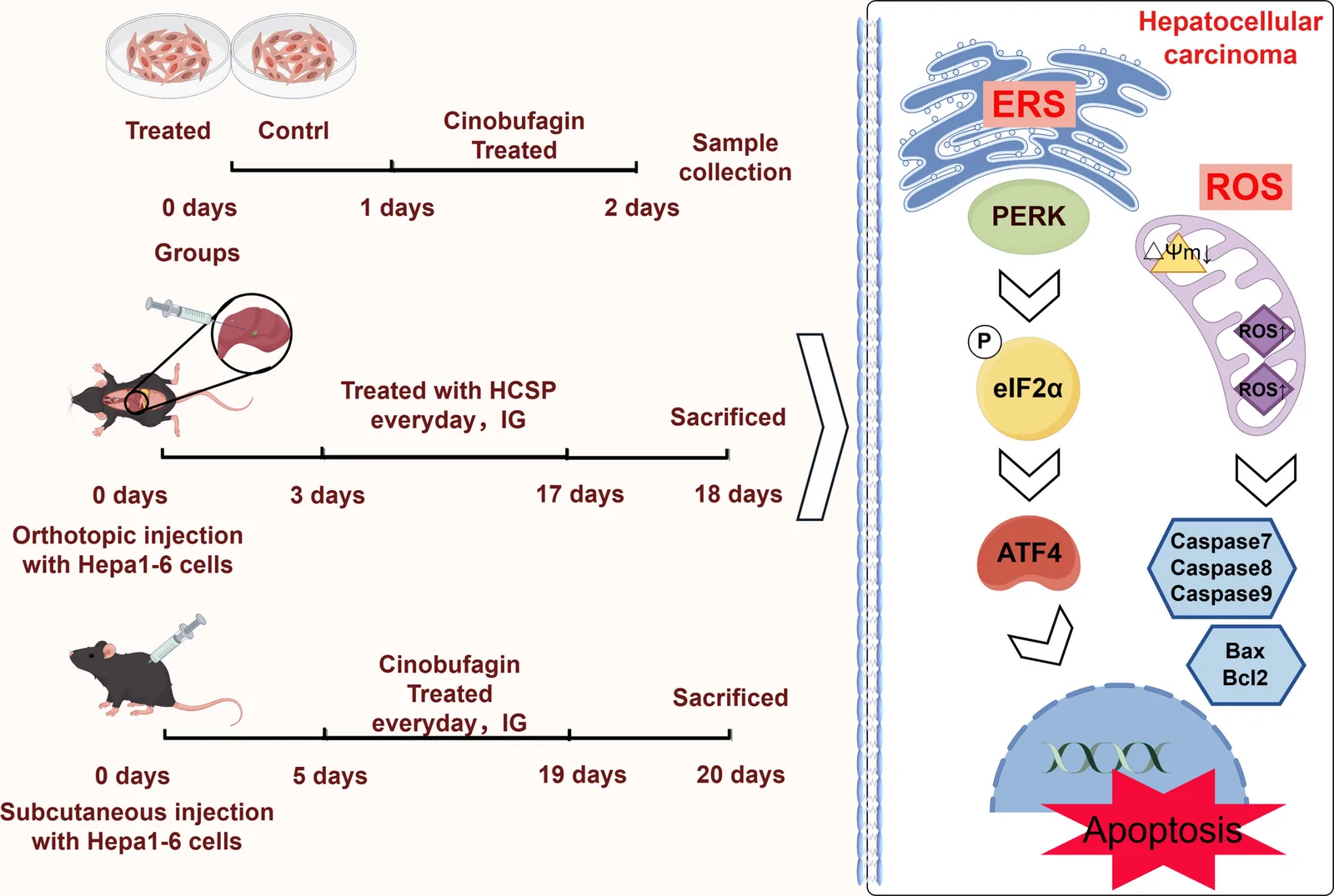

## Introduction

Hepatocellular carcinoma (HCC) is the fourth most common cancer and the second leading cause of cancer-related mortality. Globally, incident cases of HCC have increased by approximately 75% between 1990 and 2015, highlighting its rapidly growing health burden worldwide(D. Q. Huang et al. 2021). In 2020, approximately 410,000 new cases of liver cancer were diagnosed in China, accounting for 45.3% of global new diagnoses (Rumgay et al. 2022; Singh et al. 2025). HCC, which accounts for 75–85% of all primary liver cancer cases, is characterized by its high invasiveness and propensity for recurrence and metastasis, resulting in poor prognosis for affected patients (Koshy 2025; D. Xie et al. 2023). While treatment strategies such as surgical resection and transarterial chemoembolization have played a critical role in improving the survival of HCC patients, the prognosis remains limited due to the fact that most patients are diagnosed at advanced stages of the disease (Rossari et al. 2024). Therefore, identifying novel therapeutic targets and strategies is essential for improving treatment outcomes in HCC (X. Yang et al. 2024).

Emerging evidence has highlighted the crucial role of reactive oxygen species (ROS) and mitochondrial dysfunction in the initiation and progression of HCC (B. Li et al. 2019a, b). Excessive ROS production not only disrupts mitochondrial membrane potential but also activates apoptotic signaling pathways, leading to tumor cell death (Wang et al. 2024; Xing et al. 2023). Furthermore, endoplasmic reticulum (ER) stress, a key cellular response to stress, has been implicated in regulating the survival, proliferation, and apoptosis of HCC cells (Cheng et al. 2024; Khaled et al. 2022). Over the past three decades, ER stress signaling has gained considerable attention in the field of HCC research, and activating ER stress pathways may represent a promising therapeutic strategy for the treatment of HCC (Zhang et al. 2024). In hepatocellular carcinoma, basal ER stress signaling may be attenuated as part of tumor adaptive mechanisms, whereas pharmacological activation of the PERK-ATF4-CHOP pathway has been shown to promote apoptosis (Luna-Marco et al. 2023).

Huachansu, extracted from the Chinese toad (*Bufo bufo gargarizans*), possesses various pharmacological activities, including heat-clearing, detoxifying, blood-stasis-resolving, and anti-tumor effects (Meng et al. 2009; D. Tang et al. 2024; Zhan et al. 2020). It has shown significant growth inhibitory and anti-metastatic effects in several malignant tumors, including hepatocellular carcinoma (HCC) (Dai et al. 2023; Xu et al. 2023). Research has demonstrated that Huachansu exerts its anti-tumor effects through the regulation of apoptosis-related factor expression, inhibition of inflammatory responses, and increased ROS accumulation and oxidative stress (Deng et al. 2024; C. Y. Huang et al. 2023). However, the underlying molecular mechanisms of its effects in HCC remain to be fully elucidated (C. Chen et al. 2024).

In this study, we systematically evaluated the anti-tumor effects of Huachansu in both HCC cells and mouse tumor models. We focused on elucidating the molecular mechanisms by which Huachansu induces tumor cell apoptosis, particularly through mitochondrial dysfunction and endoplasmic reticulum stress signaling pathways. While both ER stress and mitochondrial apoptosis have individually been implicated in HCC progression and therapeutic responses, whether Huachansu exerts its anti-HCC effects through coordinated regulation of these two pathways remains unclear. Through in vitro and in vivo functional analyses, we explored the impact of Huachansu on mitochondrial damage and ER stress in HCC cells. Our findings are anticipated to provide new insights into the clinical potential of Huachansu and offer a basis for optimizing therapeutic strategies for HCC.

## Materials and methods

### Reagents

Huachansu (cat. no. Z34020644) was purchased from Anhui Jinchang Biochemical Co., Ltd. (Anhui, China); DMEM medium (cat. no. 11965092), Fetal bovine serum (FBS, cat. no. 16000044), and Trizol reagent (cat. no. 15596018) were purchased from Gibco (Grand Island, NY, USA); PBS buffer (cat. no. P1020) was purchased from Solarbio (Beijing, China); CCK-8 assay kit (cat. no. CK04) and Annexin V-FITC/PI dual-staining apoptosis detection kit (cat. no. KGA106) were purchased from Dojindo (Kumamoto, Japan); cDNA synthesis kit (cat. no. RR047A) and SYBR Green real-time quantitative PCR kit (cat. no. RR820A) were purchased from Takara (Dalian, China); primary antibodies for Western blot: Bax (cat. no. ab32503), Bcl-2 (cat. no. ab32124), PERK (cat. no. ab79483), and ATF4 (cat. no. ab207434) were purchased from Abcam (Cambridge, UK); caspase-8 (cat. no. 9746S) was purchased from CST (Danvers, MA, USA); secondary antibodies for immunofluorescence: Alexa Fluor 488 (cat. no. A11001) and Alexa Fluor 594 (cat. no. A11005) were purchased from Thermo Fisher (Waltham, MA, USA); RIPA lysis buffer (cat. no. P0013B), BCA protein assay kit (cat. no. P0010), and ECL detection reagent (cat. no. P0018S) were purchased from Beyotime (Shanghai, China); EDTA decalcification solution was purchased from Shanghai Biotian Biotechnology Co., Ltd. (Shanghai, China). qPCR primers for CHOP, Hsp5a, Erdj4, and Edme1 were synthesized by Sangon Biotech (Shanghai) Co., Ltd. (Shanghai, China).

### Cell culture

The human hepatocellular carcinoma cell line HepG2 was purchased from Novozan Life Sciences Co., Ltd. (Suzhou, China). The cells were authenticated by STR profiling to ensure the authenticity and stability of the cell line. Cells were cultured in MEM medium (containing NEAA, catalog no. PM150410) purchased from Wuhan Punosai Life Sciences Co., Ltd. (Wuhan, China), supplemented with 10% fetal bovine serum (catalog no. 7E602L2) from Novozan Biotech Co., Ltd. (Suzhou, China) and 1% penicillin–streptomycin solution. The cells were maintained at 37 °C in a 5% CO₂ incubator. The cells were divided into three treatment groups: (1) DMSO group, treated with an equal volume of solvent; (2) low-dose Huachansu group, treated with Huachansu at a final concentration of 48 mg/mL; (3) high-dose Huachansu group, treated with Huachansu at a final concentration of 96 mg/mL. Huachansu was dissolved in DMSO and further diluted in culture medium, with a final DMSO concentration below 0.1%. After 24 h of treatment, cell samples were collected for subsequent analysis. CCK-8 assays were performed with six biological replicates, whereas fluorescence-based assays (ROS, JC-1, and apoptosis staining) were conducted in three independent replicates.

### Establishment and treatment of orthotopic liver cancer mouse model

C57BL/6 male mice (6–8 weeks old, weighing 18–22 g) were used to establish the orthotopic liver cancer model by direct injection of 1 × 10⁶ Hepa1-6 hepatocellular carcinoma cells into the liver. The Hepa1-6 cells were cultured in the same conditions as the HepG2 cells (He et al. 2015). The mice were housed in SPF-grade experimental animal facilities, maintained at a temperature of 22 ± 2 °C, with 50 ± 10% relative humidity and a 12-h light/dark cycle. The animals had access to standard rodent chow and sterile water. All animal procedures were approved by the Animal Ethics Committee of Nanjing University of Chinese Medicine (Approval No. ACU240307) and conducted in accordance with institutional guidelines to minimize animal suffering.

Following model establishment, the mice were randomly assigned to three treatment groups (n = 6 per group): (1) model group, treated with an equal volume of physiological saline via oral gavage; (2) low-dose Huachansu group, treated with 2 g/kg Huachansu daily by oral gavage; (3) high-dose Huachansu group, treated with 4 g/kg Huachansu daily by oral gavage. Treatment was continued for 18 days post-model establishment. Huachansu was suspended in sodium carboxymethyl cellulose (CMC-Na, 0.05%) for in vivo administration. At the end of the experiment, liver tissue and serum samples were collected from the mice for subsequent analysis.

### Establishment and treatment of xenograft tumor mouse model

A xenograft tumor model was established by subcutaneously inoculating 1 × 10⁶ Hepa1-6 hepatocellular carcinoma cells into the axillary subcutaneous tissue of C57BL/6 mice (He et al. 2015). The mice were randomly divided into three treatment groups (n = 5 per group): (1) model group, treated with an equal volume of physiological saline via oral gavage; (2) low-dose Huachansu group, treated with 2 g/kg Huachansu daily by oral gavage; (3) high-dose Huachansu group, treated with 4 g/kg Huachansu daily by oral gavage. Treatment was continued for 21 days post-inoculation. Huachansu was suspended in sodium carboxymethyl cellulose (CMC-Na, 0.05%) for in vivo administration. At the end of the experiment, tumor volume and weight were recorded, and tumor tissue and serum samples were collected for subsequent analysis.

### ROS detection

The levels of reactive oxygen species (ROS) in cells were measured using a ROS fluorescence probe. After washing the samples with PBS, the cells and tissue samples were incubated with a final concentration of 10 μM ROS fluorescence probe at 37 °C for 30 min. Fluorescence intensity was observed and recorded using a fluorescence microscope (Gomes et al. 2005).

### Mitochondrial membrane potential detection

Mitochondrial membrane potential changes were assessed using the JC-1 staining method. Following PBS washing, cells were incubated with JC-1 working solution for 30 min. The red/green fluorescence ratio was observed and recorded using a fluorescence microscope (Chazotte 2011).

### TUNEL assay

Cell apoptosis in tumor tissues was detected using the TUNEL assay kit. Tissue sections were deparaffinized, permeabilized, and stained according to the manufacturer's instructions. The proportion of apoptotic cells was evaluated by observing green fluorescence-labeled apoptotic cells under a fluorescence microscope (Majtnerová and Roušar 2018).

### ELISA

Mouse serum samples were collected and analyzed for the levels of inflammatory cytokines including IL-1β and antioxidant factors including SOD using ELISA kits. The optical density (OD) was measured at 450 nm, following the manufacturer's instructions (Hornbeck 2015).

### Western blot analysis

Total protein was extracted from cells or tissues, and protein concentration was determined using the BCA method. A total of 30 μg of protein was loaded onto SDS-PAGE gels for electrophoretic separation and then transferred to PVDF membranes. Membranes were incubated with primary antibodies (Bax, Bcl-2, caspase family proteins, PERK, ATF4, eIF2α, p-eIF2α) followed by secondary antibodies. Protein bands were detected using ECL chemiluminescence and analyzed for band density using ImageJ software. The relative protein expression levels were quantified by densitometric analysis and normalized to GAPDH (Pillai-Kastoori et al. 2020).

### PCR analysis

Total RNA was extracted from cells or tissues, and cDNA was synthesized using a reverse transcription kit. The mRNA levels of CHOP, Hsp5a, Erdj4, and Edme1 were quantified using SYBR Green qPCR kit. GAPDH was used as an internal control, and relative gene expression levels were calculated using the 2^^−ΔΔCt^ method (Green and Sambrook 2018).

### IHC and immunofluorescence staining

Immunohistochemistry (IHC) was performed to detect the expression of the proliferation marker Ki67 in tumor tissues. Immunofluorescence staining was used to assess the expression levels of CRT and CHOP. Secondary antibodies conjugated to Alexa Fluor were applied for staining, and nuclei were counterstained with DAPI. Images were observed and recorded using a fluorescence microscope (Hussaini et al. 2023).

### HE, masson and sirius red staining

Paraffin-embedded liver and xenograft tumor tissue sections were stained with hematoxylin and eosin (HE) to observe histopathological changes. Masson’s trichrome and Sirius Red staining were performed to assess collagen deposition in liver tissues. Representative images were captured under a light microscope (Wick 2019).

### Tumor volume and weight measurement

In the xenograft tumor model, tumor dimensions were measured using a caliper. Tumor volume was calculated using the formula: V = length × width^2^/2, and tumor weight was measured at the experiment's endpoint (Lestini et al. 2016).

### Statistical analysis

All experimental data are expressed as mean ± standard deviation (mean ± SD). Data normality was assessed using the Shapiro–Wilk test prior to post-hoc analysis, and homogeneity of variance was evaluated using Levene’s test. Differences between groups were analyzed using one-way analysis of variance (ANOVA), and multiple comparisons between groups were performed using the LSD test after confirmation of variance homogeneity. A p-value of < 0.05 was considered statistically significant. Data analysis was conducted using SPSS version 22.0 software.

## Results

### Huachansu induces mitochondrial dysfunction and promotes apoptosis in HepG2 cells

**Fig. 1:**
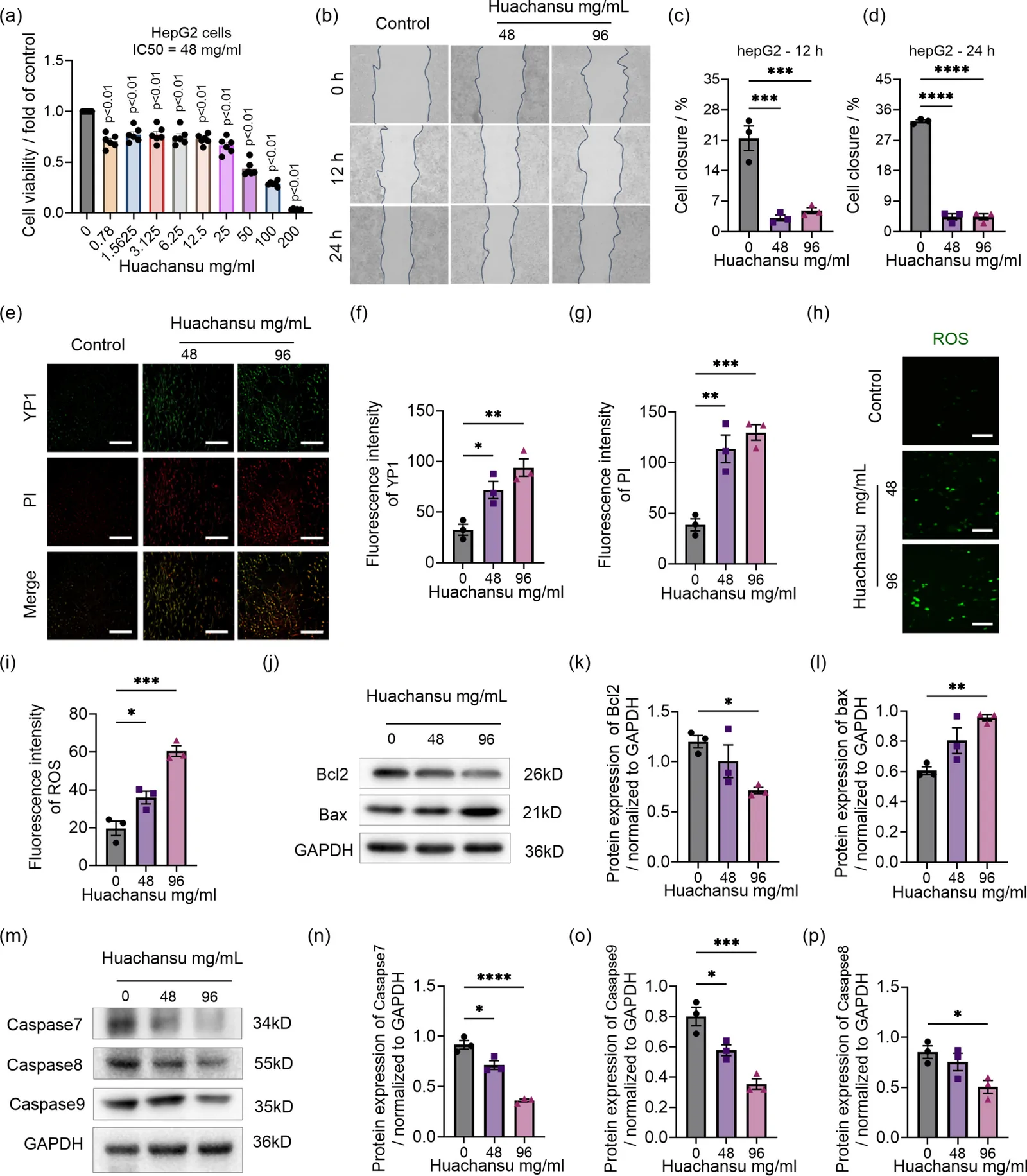
Huachansu induces mitochondrial dysfunction and apoptosis in HepG2 cells. (**a**) CCK-8 assay showing dose-dependent inhibition of HepG2 cell viability by Huachansu (0–200 mg/mL); IC₅₀ = 48 mg/mL. (**b**) Wound healing assay evaluating the migratory ability of HepG2 cells treated with 48 mg/mL and 96 mg/mL Huachansu at 12 h and 24 h. (**c**-**d**) Quantitative analysis of wound closure at 12 h (**c**) and 24 h (**d**) after treatment. (**e**) Representative images of Annexin V (YP1)/PI double staining showing apoptosis and necrosis in HepG2 cells treated with Huachansu (48 mg/mL and 96 mg/mL) for 24 h (scale bar = 100 μm). (**f**-**g**) Quantification of YP1 fluorescence intensity (**f**) and PI fluorescence intensity (**g**). (**h**) Representative fluorescence images of ROS generation in HepG2 cells treated with Huachansu (48 mg/mL and 96 mg/mL) (scale bar = 100 μm). (**i**) Quantification of intracellular ROS levels. (**j**) Western blot analysis showing the expression of pro-apoptotic protein Bax and anti-apoptotic protein Bcl-2 after 24 h treatment. (**k**-**l**) Densitometric analysis of Bax (**k**) and Bcl-2 (**l**) protein expression. (**m**) Western blot analysis of caspase-7, caspase-8, and caspase-9. (**n**-**p**) Quantification of caspase-7 (**n**), caspase-8 (**o**), and caspase-9 (**p**) protein levels

To assess the cytotoxic effect of Huachansu on liver cancer cells, we first evaluated cell viability using the CCK-8 assay across a range of concentrations (0–200 mg/mL). Huachansu exhibited a dose-dependent inhibitory effect on HepG2 cell viability, with an IC₅₀ value of 48 mg/mL **(**Fig. 1a**)**. Based on this, subsequent experiments were performed using 48 mg/mL and 96 mg/mL concentrations. Wound healing assays were used to determine the migratory ability of HepG2 cells. Huachansu significantly inhibited cell migration at both 12 h and 24 h post-treatment, as evidenced by the reduced wound closure compared to the control (Fig. 1b). Quantitative analysis revealed that the inhibitory effect was dose-dependent and became more pronounced over time (Fig. 1c-d). Apoptosis and necrosis were assessed using Annexin V (YP1)/PI double staining. Huachansu treatment significantly increased both early and late apoptosis rates, as well as necrotic cell populations, after 24 h of exposure (Fig. 1e). Quantification of fluorescence intensity showed a marked increase in YP1 and PI signals in a dose-dependent manner (Fig. 1f-g). To determine whether mitochondrial dysfunction contributed to apoptosis, we measured intracellular ROS levels. Huachansu treatment significantly increased ROS generation, as demonstrated by fluorescence microscopy and quantitative analysis, with ROS levels increasing by approximately 83% and 208% at 48 mg/mL and 96 mg/mL, respectively. (Fig. 1h-i). Western blot analysis further supported these findings. Expression of the pro-apoptotic protein Bax was significantly upregulated, while the anti-apoptotic protein Bcl-2 was downregulated following Cinobufagin treatment (Fig. 1j-l). In addition, the levels of caspase-7, caspase-8, and caspase-9 were markedly increased (Fig. 1m-p), indicating activation of both the intrinsic and extrinsic apoptotic pathways.

### Huachansu activates endoplasmic reticulum stress in HepG2 cells

**Fig. 2:**
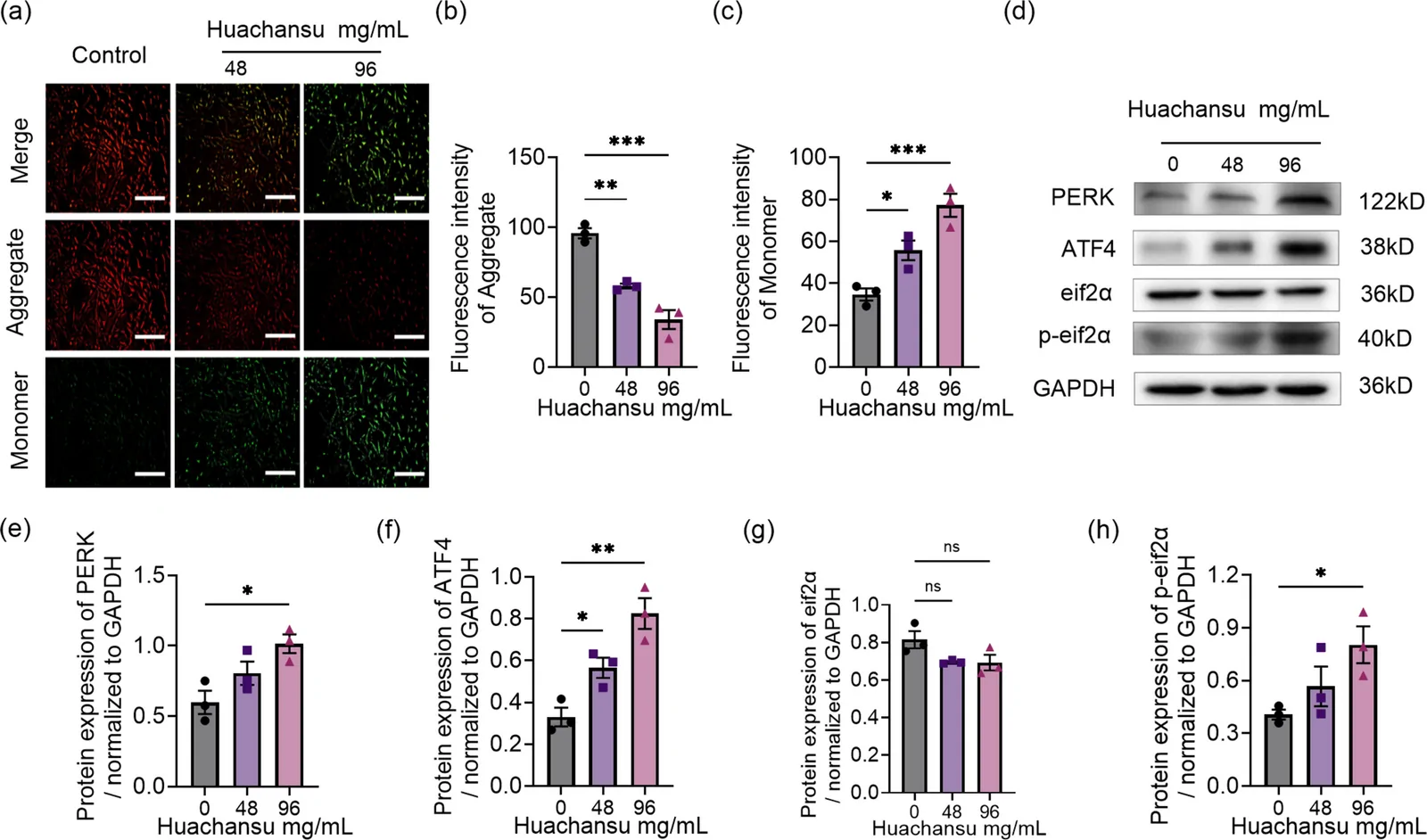
Huachansu activates endoplasmic reticulum stress in HepG2 cells. (**a**) Representative images of JC-1 staining showing changes in mitochondrial membrane potential in HepG2 cells treated with Huachansu (48 mg/mL and 96 mg/mL) for 24 h (scale bar = 100 μm). (**b**-**c**) Quantification of JC-1 aggregate (red) fluorescence (b) and monomer (green) fluorescence (**c**). (**d**) Western blot analysis showing the expression of PERK, ATF4, eIF2α, and phosphorylated eIF2α (p-eIF2α) after Huachansu treatment. (**e**–**h**) Densitometric analysis of PERK (**e**), ATF4 (**f**), eIF2α (**g**), and p-eIF2α (**h**) protein levels

To determine whether Huachansu induces endoplasmic reticulum (ER) stress in liver cancer cells, we examined changes in mitochondrial membrane potential and the expression of ER stress-related signaling proteins. JC-1 staining revealed a significant decrease in mitochondrial membrane potential following treatment with Huachansu at 48 mg/mL and 96 mg/mL for 24 h, as evidenced by decreased red (aggregate) fluorescence and increased green (monomer) fluorescence (Fig. 2a). Quantitative analysis showed a dose-dependent decrease in aggregate (red) fluorescence and a corresponding increase in monomer (green) fluorescence, consistent with mitochondrial depolarization (Fig. 2b-c). To assess ER stress activation, Western blot analysis was performed to evaluate the expression of PERK, ATF4, eIF2α, and phosphorylated eIF2α (p-eIF2α). Huachansu treatment markedly upregulated PERK and ATF4 expression and significantly enhanced the phosphorylation of eIF2α in a dose-dependent manner, while total eIF2α levels remained unchanged (Fig. 2d). Densitometric quantification further confirmed that the 96 mg/mL treatment group exhibited the strongest ER stress response (Fig. 2e-h).

### Huachansu reduces tumor burden and improves liver function in an orthotopic hepatocellular carcinoma model

**Fig. 3:**
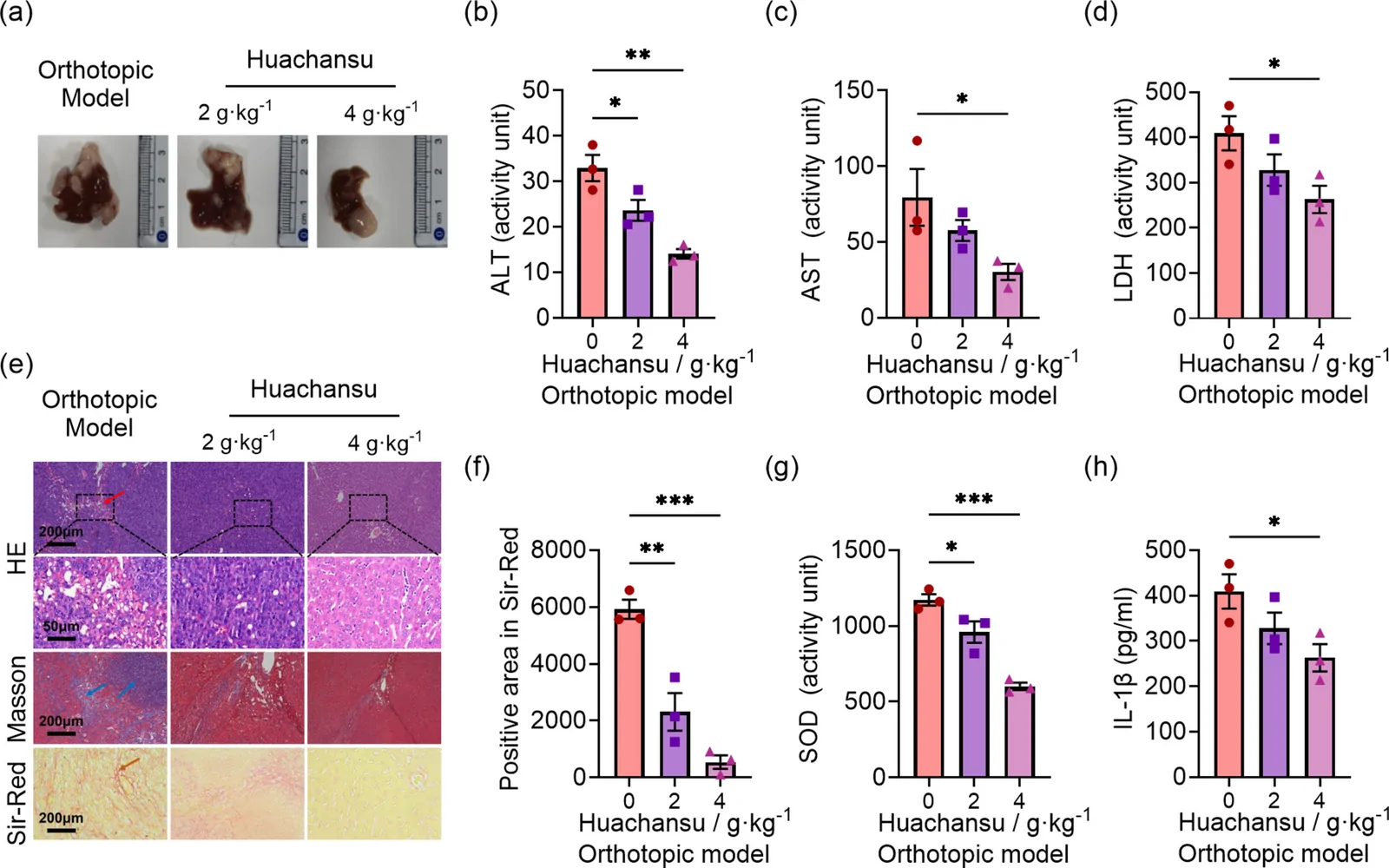
Huachansu reduces tumor burden in the orthotopic liver cancer mouse model. (**a**) Representative images of liver tissue showing a significant reduction in tumor number in Huachansu-treated mice (2 g/kg and 4 g/kg). (**b**-**d**) Serum ALT (**b**), AST (**c**), and LDH (**d**) levels in each group. (**e**) HE, Masson, and Sirius Red staining showing improved pathological morphology and reduced collagen deposition in liver tissue after Huachansu treatment. Red arrows indicate fibrotic regions in liver tissue at low magnification; blue arrows indicate collagen deposition in Masson staining; red arrows indicate collagen-positive areas in Sirius Red staining. (**f**) Quantification of Sirius Red-positive area in liver tissue. (**g**) Serum SOD levels in Huachansu-treated mice. (**h**) ELISA analysis of serum IL-1β levels in each group

To investigate the antitumor efficacy of Huachansu in vivo, we established an orthotopic liver cancer model by injecting Hepa1-6 cells directly into the liver of C57BL/6 mice. Huachansu treatment at doses of 2 g/kg and 4 g/kg for 18 consecutive days significantly reduced visible tumor burden in the liver compared to the untreated model group (Fig. 3a). Serum biochemical analysis showed that the levels of alanine aminotransferase (ALT), aspartate aminotransferase (AST), and lactate dehydrogenase (LDH) were significantly decreased in the Huachansu-treated groups (Fig. 3b-d), indicating an improvement in hepatic injury. Histological examination of liver tissue demonstrated that Huachansu notably ameliorated tumor-associated histopathological changes, as observed by HE staining. In addition, Masson’s trichrome and Sirius Red staining revealed that collagen deposition was markedly reduced following treatment (Fig. 3e). Quantification of Sirius Red-positive areas further confirmed the antifibrotic effect of Huachansu (Fig. 3f). Furthermore, Huachansu significantly reduced serum levels of the antioxidant enzyme superoxide dismutase (SOD), suggesting altered oxidative stress status (Fig. 3g). Concurrently, enzyme-linked immunosorbent assay (ELISA) analysis revealed a significant reduction in the inflammatory cytokine interleukin-1β (IL-1β) (Fig. 3h).

### Huachansu inhibits tumor proliferation by promoting apoptosis in an orthotopic hepatocellular carcinoma model

**Fig. 4:**
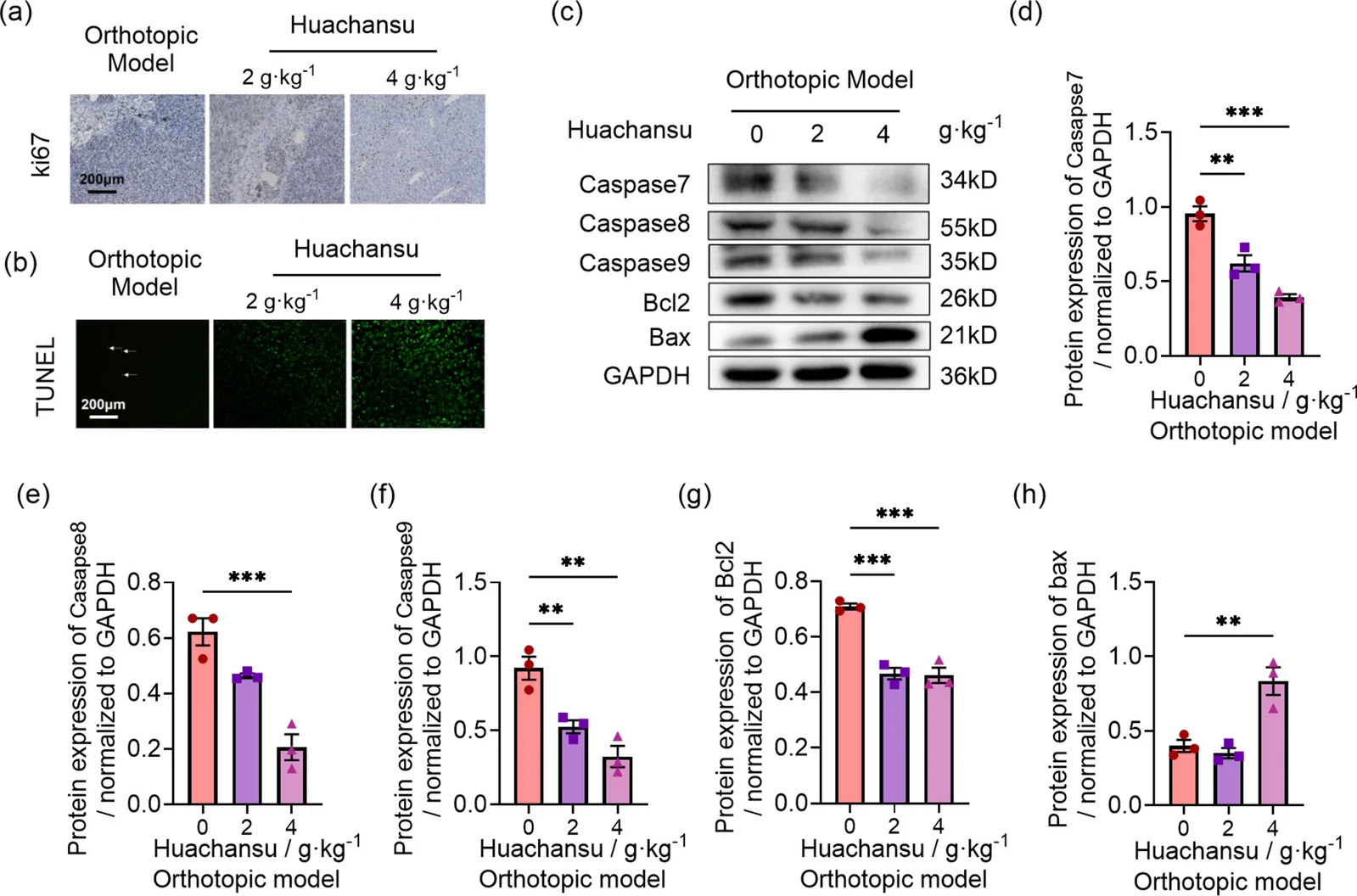
Huachansu inhibits tumor proliferation by promoting apoptosis in an orthotopic hepatocellular carcinoma model. (**a**) Immunohistochemical staining of Ki67 showing reduced proliferation in orthotopic liver tumor tissues from Huachansu-treated mice (2 g/kg and 4 g/kg). Black arrows indicate representative positive staining. (**b**) TUNEL assay indicating increased apoptosis in tumor tissues. White arrows indicate representative TUNEL-positive areas. (**c**) Western blot analysis of caspase-7, caspase-8, caspase-9, Bax, and Bcl-2 protein expression. (**d**-**h**) Densitometric quantification of caspase-7 (**d**), caspase-8 (**e**), caspase-9 (**f**), Bax (**g**), and Bcl-2 (**h**)

To further elucidate the mechanism by which Huachansu exerts its antitumor effects in *vivo*, we assessed cell proliferation and apoptosis in tumor tissues of the orthotopic HCC mouse model. The proliferation marker Ki67 was markedly reduced in the Huachansu-treated groups (2 g/kg and 4 g/kg), compared to the model group (Fig. 4a). To evaluate apoptosis, TUNEL staining was performed and demonstrated a clear increase in the proportion of apoptotic cells in Huachansu-treated tumors (Fig. 4b). Western blot analysis of apoptosis-related proteins showed that Huachansu treatment markedly reduced the expression of caspase-7, caspase-8, and caspase-9, accompanied by upregulation of pro-apoptotic Bax and downregulation of anti-apoptotic Bcl-2, suggesting modulation of apoptotic pathways (Fig. 4c). Densitometric quantification of these proteins confirmed that the apoptotic response was more pronounced in the 4 g/kg group (Fig. 4d-h).

### Huachansu activates endoplasmic reticulum stress in an orthotopic hepatocellular carcinoma model

**Fig. 5:**
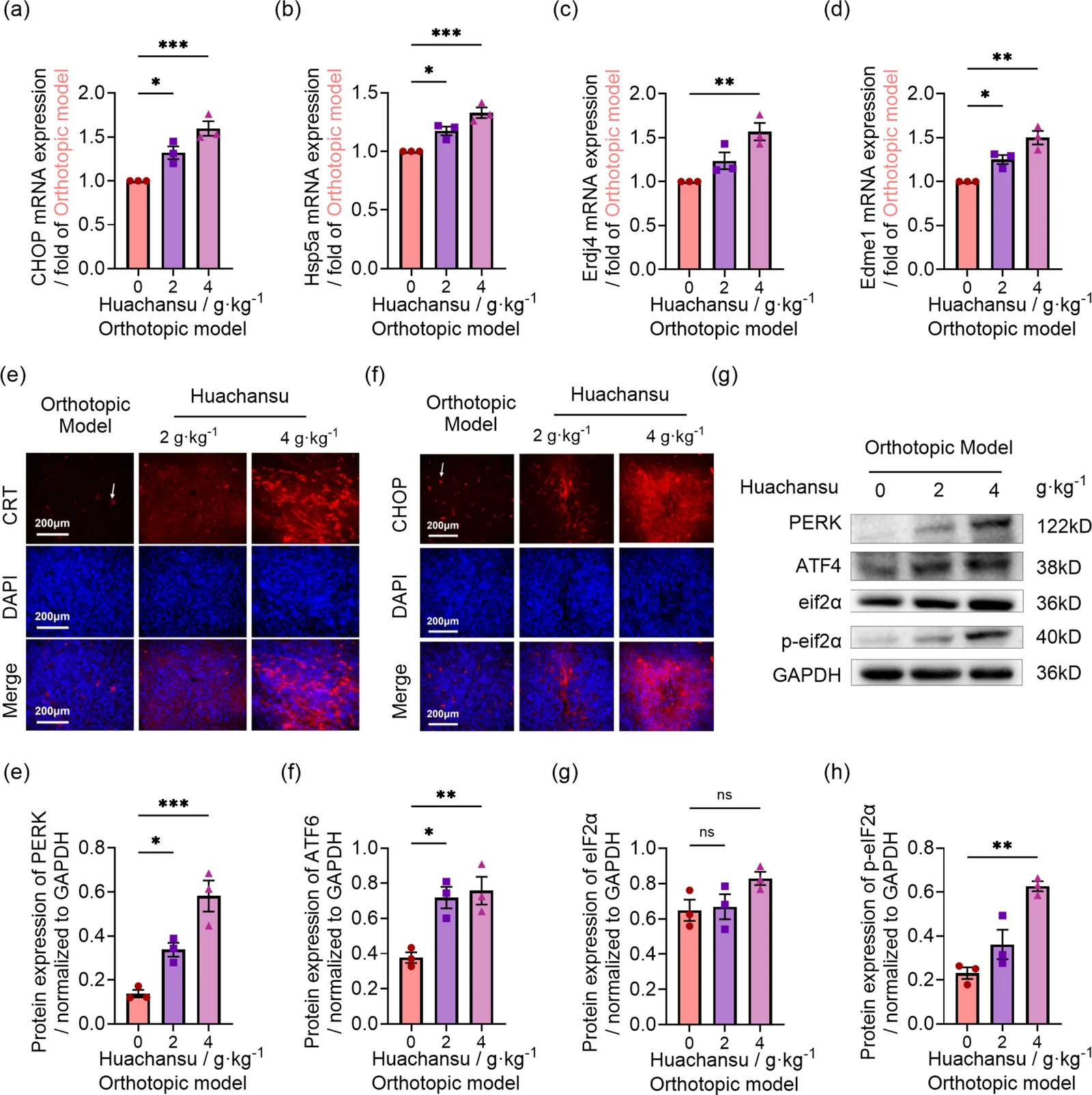
Huachansu activates endoplasmic reticulum stress in the orthotopic liver cancer model. (**a**-**d**) qPCR analysis showing increased mRNA expression of CHOP (**a**), Hspa5 (**b**), Erdj4 (**c**), and Edem1 (**d**) in tumor tissues from Huachansu-treated mice. (**e**–**f**) Immunofluorescence staining showing elevated CRT (**e**) and CHOP (**f**) expression in tumor tissues. White arrows indicate representative positive staining areas. (**g**) Western blot analysis of ER stress-related proteins PERK, ATF4, eIF2α, and p-eIF2α. (**h**–**k**) Densitometric quantification of PERK (**h**), ATF4 (**i**), eIF2α (**j**), and p-eIF2α (**k**) protein expression levels

To investigate whether Huachansu activates ER stress in vivo, we examined ER stress markers in tumor tissues from the orthotopic HCC mouse model. Quantitative real-time PCR analysis showed that Huachansu treatment significantly increased the mRNA levels of CHOP, Hspa5 (GRP78), Erdj4, and Edem1 compared to the model group (Fig. 5a-d), indicating transcriptional activation of ER stress-associated genes. Immunofluorescence staining further revealed markedly increased expression of calreticulin (CRT) and CHOP in Huachansu-treated tumor tissues (Fig. 5e-f). Consistently, Western blot analysis demonstrated significant upregulation of ER stress pathway proteins, including PERK, ATF4, eIF2α, and phosphorylated eIF2α (p-eIF2α) (Fig. 5g). Densitometric quantification confirmed a dose-dependent increase in protein expression, particularly in the high-dose group (Fig. 5h-k).

### Huachansu suppresses tumor growth in a hepatocellular carcinoma xenograft model

**Fig. 6:**
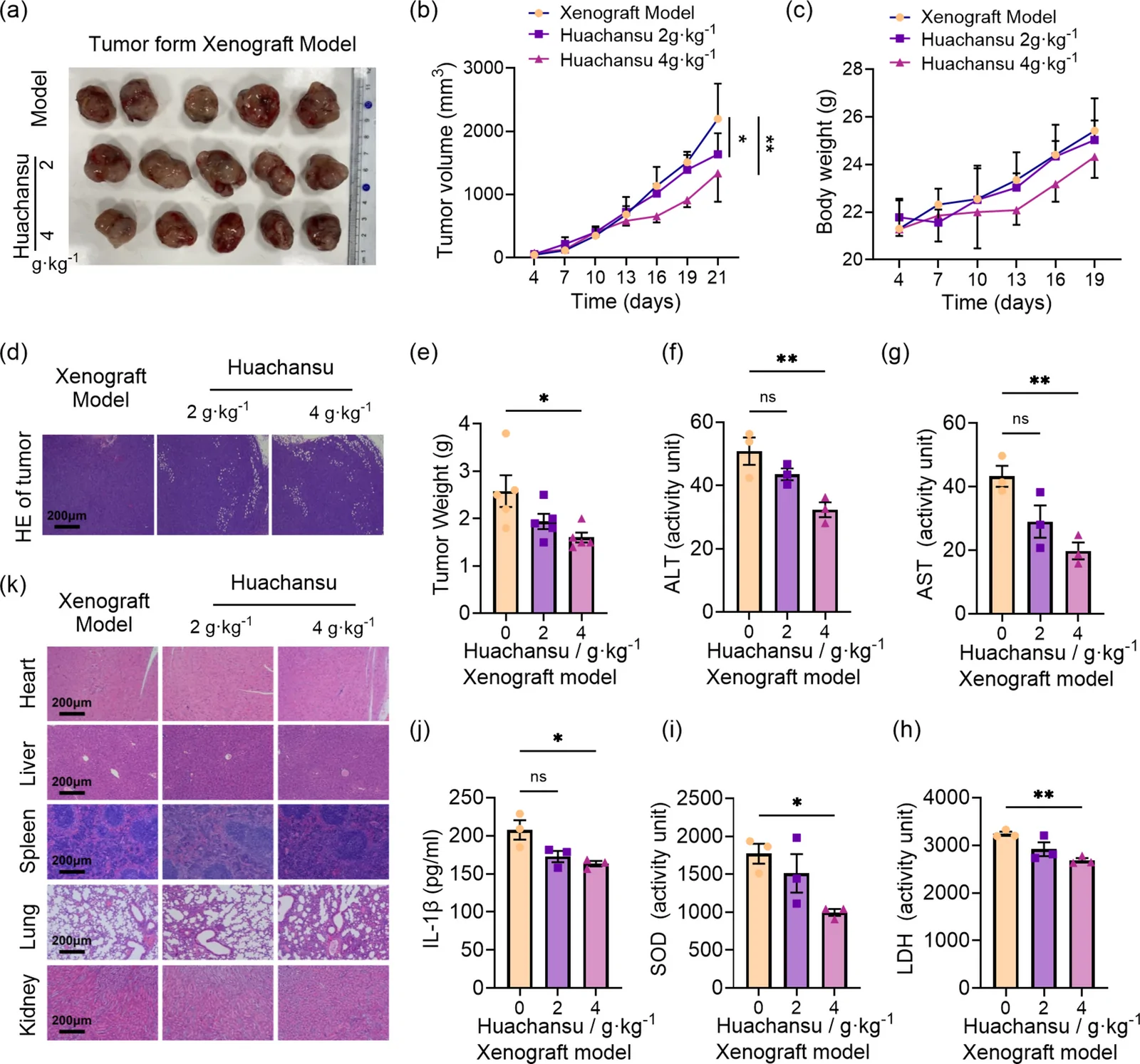
Huachansu exhibits antitumor effects in the xenograft tumor model. (**a**) Representative images of tumors collected from each group at the endpoint of the experiment. (**b**) Tumor volume progression recorded every 3 days for 21 days. (**c**) Body weight of mice monitored every 3 days during treatment. (**d**) HE staining of tumor tissues showing morphological improvement after Huachansu treatment. (**e**) Tumor weight statistics at the study endpoint. (**f**–**h**) Serum levels of ALT (f), AST (**g**), and LDH (**h**) in each group. (**i**) Serum SOD levels. (**j**) ELISA analysis of serum IL-1β levels. (**k**) HE staining of major organs (heart, liver, spleen, lungs, kidneys) showing no evident histological abnormalities

To complement the orthotopic model findings, we employed a hepatocellular carcinoma xenograft model by subcutaneously inoculating Hepa1-6 cells into C57BL/6 mice. Huachansu administration at doses of 2 g/kg and 4 g/kg significantly inhibited tumor growth, as evidenced by the reduction in tumor volume over the 21-day observation period, with reductions of approximately 25% and 39% in the low- and high-dose groups, respectively (Fig. 6a-b). Throughout the treatment course, no significant changes in body weight were observed (Fig. 6c), suggesting good overall tolerability. Histological analysis of tumor tissues revealed improved histological architecture and reduced tumor cell density following Huachansu treatment, as assessed by HE staining (Fig. 6d). At the study endpoint, tumor weight was also markedly lower in the treatment groups compared to the model group (Fig. 6e), indicating a dose-dependent therapeutic effect. Serum biochemical assays further supported the protective effects of Huachansu. The levels of ALT, AST, and LDH were significantly reduced in Huachansu-treated mice, suggesting alleviated liver injury and improved systemic condition (Fig. 6f-h). Moreover, Huachansu reduced serum SOD levels, indicating alterations in antioxidant status (Fig. 6i), and reduced inflammatory response, as shown by decreased serum IL‑1β levels (Fig. 6j). Importantly, HE staining of major organs—including the heart, liver, spleen, lungs, and kidneys—revealed no noticeable histopathological abnormalities in any group, indicating good systemic safety (Fig. 6k).

### Huachansu inhibits tumor proliferation by promoting apoptosis in a hepatocellular carcinoma xenograft model

**Fig. 7:**
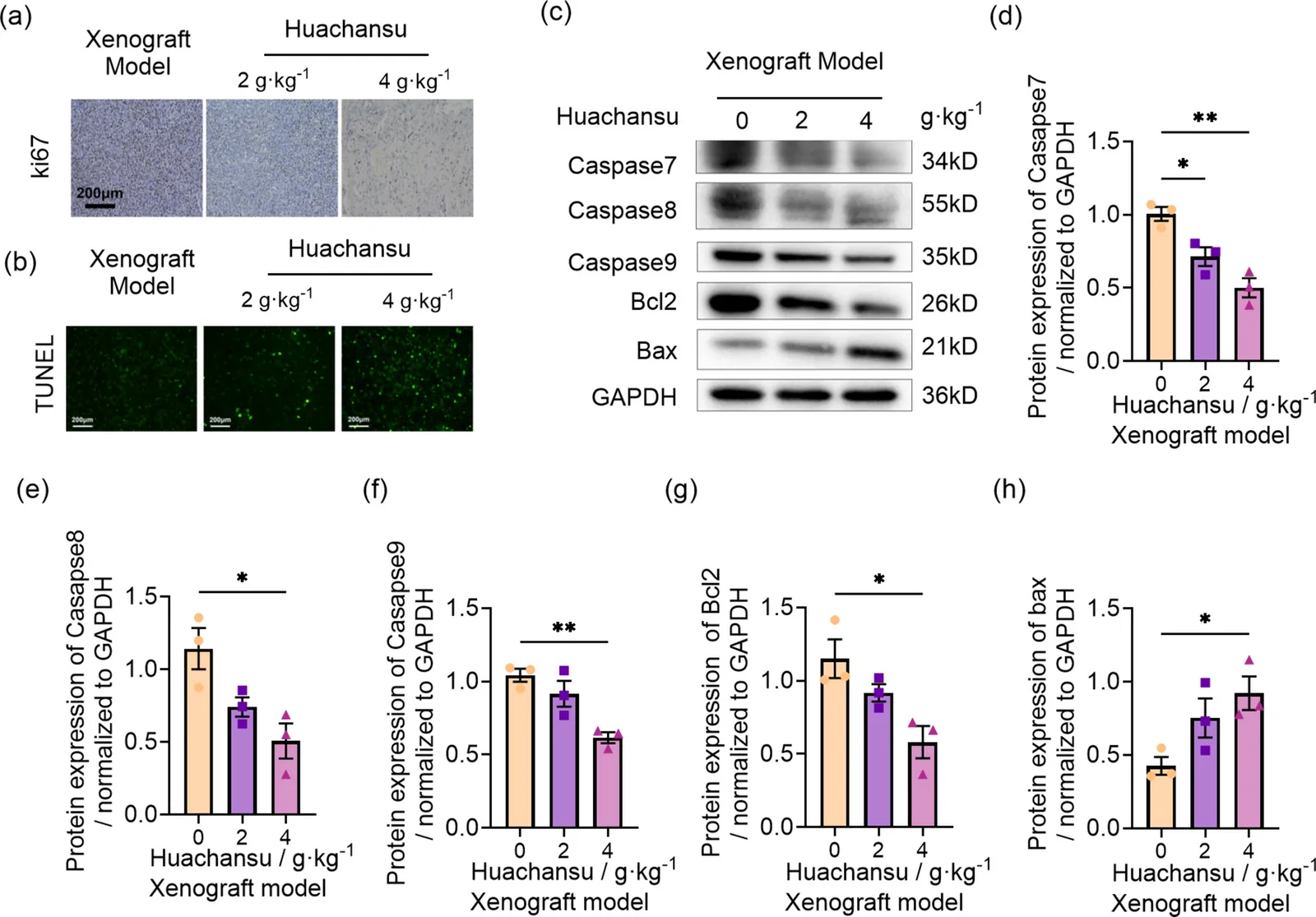
Huachansu inhibits tumor proliferation by promoting apoptosis in the hepatocellular carcinoma xenograft model. (**a**) Immunohistochemical staining of Ki67 showing reduced proliferation in xenograft tumor tissues from Huachansu-treated mice (2 g/kg and 4 g/kg). (**b**) TUNEL assay indicating increased apoptosis in tumor tissues. (**c**) Western blot analysis of caspase-7, caspase-8, caspase-9, Bax, and Bcl-2 protein expression. (**d**-**h**) Densitometric quantification of caspase-7 (**d**), caspase-8 (**e**), caspase-9 (**f**), Bax (**g**), and Bcl-2 (**h**)

Consistent with the orthotopic model, Huachansu also inhibited tumor proliferation in the xenograft model. Immunohistochemical staining of Ki67 revealed a marked decrease in proliferating tumor cells in the Huachansu-treated groups (2 g/kg and 4 g/kg) compared to the model group (Fig. 7a). These findings indicate that Huachansu effectively suppresses tumor cell proliferation in the xenograft tumors. To assess apoptosis, TUNEL staining demonstrated a significant increase in apoptotic cells in tumors from Huachansu-treated mice, confirming the pro-apoptotic effect of the treatment (Fig. 7b). Western blot analysis further revealed that Huachansu treatment reduced the expression of caspase-7, caspase-8, and caspase-9. In addition, Huachansu administration markedly enhanced the expression of pro-apoptotic Bax while reducing anti-apoptotic Bcl-2 levels (Fig. 7c), suggesting that both intrinsic and extrinsic apoptotic pathways were affected. Densitometric quantification showed that these apoptotic markers were significantly altered in a dose-dependent manner, with the most pronounced effects observed in the 4 g/kg group (Fig. 7d-h).

### Huachansu activates endoplasmic reticulum stress in the xenograft tumor model

**Fig. 8:**
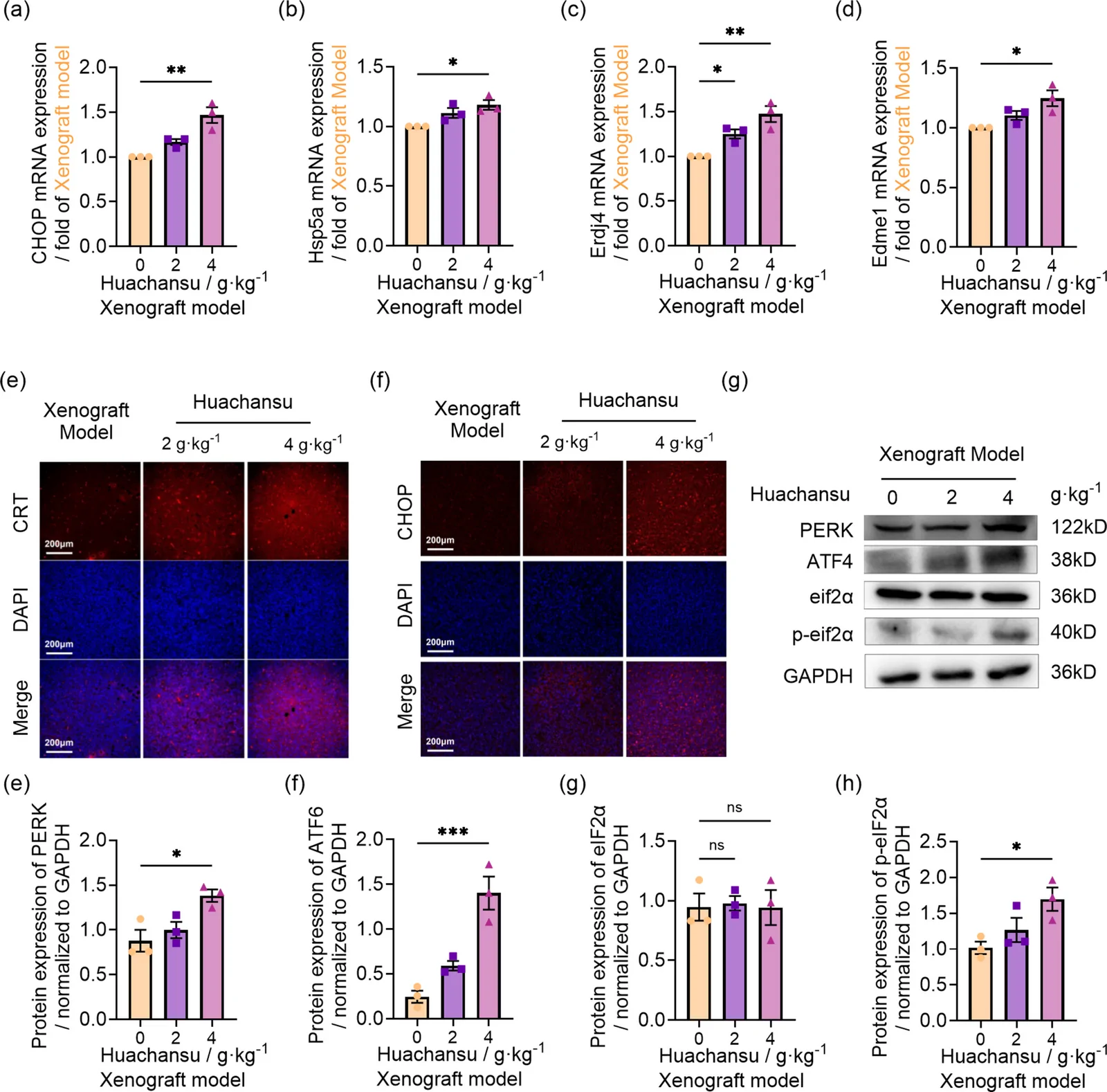
Huachansu activates endoplasmic reticulum stress in the xenograft tumor model. (**a**-**d**) qPCR analysis showing increased mRNA expression of CHOP (**a**), Hspa5 (**b**), Erdj4 (**c**), and Edem1 (**d**) in tumor tissues from Huachansu-treated mice. (**e**–**f**) Immunofluorescence staining showing elevated CRT (**e**) and CHOP (**f**) expression in xenograft tumors. (**g**) Western blot analysis of PERK, ATF4, eIF2α, and p-eIF2α. (**h**–**k**) Densitometric quantification of PERK (**h**), ATF4 (**i**), eIF2α (**j**), and p-eIF2α (**k**) protein expression levels

To examine whether Huachansu also induces ER stress in the xenograft tumor model, we assessed the expression of classical ER stress markers. qPCR analysis showed significantly elevated mRNA levels of CHOP, Hspa5, Erdj4, and Edem1 in the Huachansu-treated groups compared to the model group (Fig. 8a-d). Immunofluorescence staining further confirmed increased protein expression of CRT and CHOP in xenograft tumor tissues (Fig. 8e-f). Western blot analysis revealed upregulated expression of PERK, ATF4, eIF2α, and p-eIF2α following Huachansu treatment (Fig. 8g). Densitometric quantification demonstrated a dose-dependent enhancement of ER stress pathway activation, with the highest expression observed in the 4 g/kg group (Fig. 8h-k).

## Discussion

In this study, we comprehensively evaluated the anti-HCC effects of Huachansu through both in vitro and in vivo experiments, uncovering its potential mechanisms of action. Our results demonstrated that Huachansu induces apoptosis in liver cancer cells primarily through mitochondrial dysfunction and activation of the endoplasmic reticulum (ER) stress pathway. Specifically, Huachansu significantly activated the PERK-ATF4 signaling pathway, along with the upregulation of key downstream molecules such as CHOP and Erdj4, thereby triggering apoptosis via ER stress. Additionally, Huachansu increased reactive oxygen species (ROS) production, which led to mitochondrial membrane depolarization and further amplified apoptotic signaling by upregulating Bax, downregulating Bcl-2, and activating caspase family proteins. In vivo, Huachansu demonstrated significant anti-tumor effects in both orthotopic and xenograft liver cancer mouse models. Treatment with Huachansu resulted in a marked reduction in tumor size and weight, as well as improved liver function, as indicated by decreased ALT, AST, and LDH levels. Furthermore, Huachansu decreased collagen deposition and suppressed inflammatory markers, which contributed to a more favorable tumor microenvironment. These findings provide compelling preclinical evidence supporting the therapeutic potential of Huachansu in the treatment of HCC, and lay a foundation for its further clinical development.

In our study, Huachansu was found to significantly induce mitochondrial dysfunction and decrease mitochondrial membrane potential in HepG2 cells by increasing ROS levels, which in turn activated apoptosis-related signaling pathways (Du et al. 2024). Specifically, Huachansu upregulated Bax expression, downregulated Bcl-2 expression, and increased the level of caspase-7, caspase-8, and caspase-9. Mitochondrial damage is a well-established mechanism underlying the anticancer effects of many drugs, including doxorubicin and cisplatin, which inhibit HCC cell proliferation by generating ROS and inducing mitochondrial dysfunction (Z. Tang et al. 2021; T. Xie et al. 2024). Previous studies on Huachansu in the context of liver cancer have shown that it significantly affects VEGF and AKT signaling pathways (Deng et al. 2024; Liao et al. 2024). Furthermore, the activation of the PERK-eIF2α-ATF4-CHOP pathway during ER stress has been identified as a key factor in the induction of HCC apoptosis by natural compounds, such as cinobufagin and other bufadienolides (D. Li et al. 2019a, b; Lin et al. 2024; Luna-Marco et al. 2023). In line with this, our results demonstrate that Huachansu activates the PERK-ATF4 pathway, enhances the phosphorylation of eIF2α, and promotes ER stress. As a consequence, HepG2 cells treated with Huachansu showed increased expression of ER stress markers, including CHOP, Hsp5a, and Erdj4. This pattern suggests that Huachansu-induced ER stress is not merely adaptive but shifts toward a pro-apoptotic response, in which CHOP upregulation likely serves as a critical mediator of ER stress-associated cell death. Although ER stress-related markers such as Hspa5 (GRP78), Erdj4, and Edem1 are typically associated with adaptive or cytoprotective responses, their concurrent upregulation together with CHOP in the present study suggests a transition toward excessive ER stress, which is more likely to promote apoptosis rather than confer tumor cell protection.

To determine whether Huachansu exerts its anti-HCC effects through the activation of the PERK-ATF4 signaling pathway, we further investigated its actions using both orthotopic liver cancer and xenograft mouse models (Rozpedek et al. 2016). In the orthotopic liver cancer model, Huachansu reduced the tumor burden in a dose-dependent manner and significantly improved serum levels of ALT, AST, and LDH. It also decreased the expression of pro-inflammatory cytokines, suggesting that Huachansu not only exerts direct anti-HCC effects but may also enhance its therapeutic potential by modulating the tumor microenvironment (Peng et al. 2024). Histological analysis revealed that Huachansu alleviated liver tissue damage and reduced collagen deposition, indicating its potential in mitigating liver fibrosis (Luangmonkong et al. 2023). Moreover, the significant decrease in serum SOD levels further suggests that Huachansu may increase oxidative stress, thereby enhancing tumor cell sensitivity to oxidative damage.

Notably, previous studies have attributed the anticancer effects of cinobufagin and related bufadienolides to multiple alternative mechanisms, including AKT/ERK modulation, PI3K–AKT–mTOR signaling suppression, metabolic reprogramming, and cuproptosis-related pathways. Recent studies have highlighted the potential of Cinobufagin, a major active component derived from Huachansu, in modulating key pathways involved in liver cancer progression (Feng et al. 2022; Xu et al. 2023; A. Yang et al. 2022; A. L. Yang et al. 2021). Consistent with these findings, our study demonstrates that Huachansu not only improves liver function and suppresses fibrosis in vivo but also induces apoptosis in HCC cells through the activation of ER stress. This aligns with previous reports showing that cinobufagin, as one of the principal bioactive constituents of Huachansu, effectively inhibits HCC cell proliferation and migration by activating apoptosis and modulating the AKT and ERK pathways (Feng et al. 2022). Furthermore, the pro-apoptotic effects of Huachansu observed in our study—evidenced by TUNEL assay, immunohistochemical Ki67 reduction, and Western blot analysis of apoptotic markers Bax, caspase-8, and caspase-9—cinobufagin, one of the major active constituents of Huachansu, induces protective autophagy and apoptosis in HCC cells by suppressing the PI3K-AKT-mTOR pathway (Xu et al. 2023). Notably, as a bioactive component present in Huachansu, cinobufagin's ability to enhance cuproptosis-related gene expression and disrupt copper homeostasis provides an additional layer of evidence for its efficacy in targeting cancer cell death mechanisms (AmeliMojarad et al. 2024).

Our findings also revealed that Huachansu activates ER stress markers such as CHOP, Hsp5a, and Erdj4, further confirming its activation of the PERK-ATF4 signaling pathway, a mechanism increasingly recognized in hepatocellular carcinoma progression and therapeutic response (Y. L. Chen et al. 2025; Daverkausen-Fischer et al. 2021). This complements emerging evidence that cinobufagin, a principal bioactive constituent of Huachansu, exerts antitumor effects by interfering with metabolic reprogramming, including lipid, amino acid, carbohydrate, and nucleotide metabolism, which are hallmarks of HCC progression (A. Yang et al. 2023). Additionally, as an active component present in Huachansu, cinobufagin’s induction of DNA damage, likely via proteasomal degradation of key enzymes such as thymidylate synthase (A. Yang et al. 2022), underscores its multifaceted approach to inhibiting tumor growth.

In the xenograft liver cancer mouse model, Huachansu also demonstrated a dose-dependent antitumor effect, reducing both the volume and weight of the liver cancer cell-derived xenografts. Similar to the orthotopic liver cancer model, Huachansu induced a significant enhancement of apoptotic signaling in the xenograft model through the regulation of Bax and Bcl-2 expression, as well as the activation of caspase family proteins. Moreover, the expression of ER stress markers, such as CHOP, Hsp5a, and CRT, was notably upregulated in the Huachansu treatment group. In addition, it should be noted that CRT is primarily localized in the endoplasmic reticulum but can translocate to the cell membrane or extracellular space under stress conditions. In the present study, the immunofluorescence signals mainly reflect intracellular CRT expression, and thus the results should be interpreted with consideration of its subcellular localization. Importantly, this study also assessed the potential toxicity of Huachansu to major organs at the current dose in the xenograft model. HE staining results revealed that Huachansu did not induce significant pathological damage to key organs, including the heart, liver, spleen, lungs, and kidneys, suggesting its safety in the treatment of HCC. These findings are consistent with previous reports demonstrating that bioactive constituents of Huachansu exhibit sustained antitumor potential, supporting its possible application as a long-term anticancer agent (Dai et al. 2023).

However, there are several limitations to this study. First, while both ER stress and mitochondrial apoptotic pathways were significantly activated in this research, the coupling and cross-regulation between these two pathways in response to Huachansu remain unclear. Second, although the effective dose and potential toxicity of Huachansu in vivo have been preliminarily assessed, its combination with other anticancer therapies and its broader molecular interaction networks require further investigation. Future studies should focus on dissecting the interplay between ER stress and mitochondrial function, and further clinical research is needed to validate the therapeutic potential and safety of Huachansu, to facilitate its clinical translation for the treatment of HCC. In addition, although preliminary cytotoxicity screening was performed in both human (HepG2) and murine (Hepa1-6) HCC cells, mechanistic studies were conducted primarily in HepG2 cells due to their higher sensitivity and closer relevance to human tumor biology.

## Conclusion

In conclusion, this study demonstrates that Huachansu exerts significant anti-hepatocellular carcinoma effects both in vitro and in vivo. Mechanistically, Huachansu induces tumor cell apoptosis through coordinated activation of mitochondrial dysfunction and the PERK–eIF2α–ATF4–CHOP-mediated ER stress pathway. In addition, Huachansu modulates oxidative stress status and tumor microenvironment-related factors, further contributing to its antitumor activity. These findings provide mechanistic insights into the anticancer effects of Huachansu and support its potential as a therapeutic agent for hepatocellular carcinoma.

## Data Availability

All data supporting the findings of this study are available within the paper and its Supplementary Information.
